# Corneal topometric, aberrometric and biomechanical parameters in mucopolysaccharidosis patients

**DOI:** 10.1371/journal.pone.0218108

**Published:** 2019-06-27

**Authors:** Joanna Wasielica-Poslednik, Alexander K. Schuster, Giuseppe Politino, Susanne Marx-Gross, Katharina Bell, Norbert Pfeiffer, Susanne Pitz

**Affiliations:** 1 Department of Ophthalmology, University Medical Center Mainz, Johannes Gutenberg- University Mainz, Mainz, Germany; 2 Bürgerhospital, Frankfurt am Main, Germany; Bascom Palmer Eye Institute, UNITED STATES

## Abstract

**Aims:**

To report corneal topometric and aberrometric values in mucopolysaccharidosis (MPS) and to investigate their correlation with biomechanical corneal parameters.

**Methods:**

One randomly chosen eye of 20 MPS patients with no to moderate corneal clouding and one eye of 23 healthy controls with comparable age were prospectively included into this study. Corneal surface regularity was assessed by index of surface variance (ISV), -vertical asymmetry (IVA), -height asymmetry (IHA), -height decentration (IHD); keratoconus index (KI), central keratoconus index (CKI) and Zernike indices of anterior and posterior corneal surface using Scheimpflug imaging (Pentacam). Corneal resistance factor (CRF) and corneal hysteresis (CH) were assessed by Ocular Response Analyzer. Statistical analyses were performed using Mann-Whitney-Test and Spearman Correlation Coefficients.

**Results:**

IVA, ISV, IHD, IHA, but not KI and CKI were significantly higher in MPS patients compared to age matched healthy controls. Spherical aberration and asphericity coefficients either at the anterior or at the posterior corneal surface differed significantly between both groups. The grade of the MPS-associated corneal opacity correlated significantly with ISV (rho = 0.52), IVA (rho = 0.54), IHA (rho = 0.57) and IHD (rho = 0.48). Density of the MPS-affected corneas correlated significantly with ISV (rho = 0.52), IVA (rho = 0.72), IHA (rho = 0.57), IHD (rho = 0.69), 3rd order horizontal trefoil aberration at the posterior (rho = 0.62) and anterior surface (rho = 0.48) as well as with CH (rho = 0.55) and CRF (rho = 0.57). Spherical aberration at the back surface correlated with CRF and CH in MPS and in healthy controls.

**Conclusions:**

This is the first study analyzing shape of the corneal surface in MPS patients. Topometric indices of corneal asymmetry are significantly increased and correlate with MPS-related corneal opacity and density. Spherical aberration and asphericity coefficient at the front and at the back corneal surface differ significantly between MPS and healthy controls.

## Introduction

Progressive stromal corneal clouding is the most common ocular pathology in patients suffering from mucopolysaccharidosis (MPS). Irregularities of the corneal surface and an increase in total higher order aberrations (HOAs) were reported in patients suffering from hereditary corneal dystrophies [1]. The results suggested that altered HOAs together with stromal opacity degrade visual function. As corneal clouding appears in MPS and visual acuity can be impaired, we aimed to analyze the shape of the corneal surface and HOAs in MPS patients. To the best of our knowledge this is the first evaluation of these parameters in MPS and this study could help unravel the cause of visual impairment in these patients.

Mucopolysaccharidoses, which belong to the lysosomal storage disease family, are caused by the deficiency or malfunction of enzymes responsible for dispartment of glycosaminoglycans (GAG). The intralysosomal accumulation of interstage GAG-products induce characteristic organ damage with consecutive multi-morbidity and reduced life expectancy [2]. With the cumulative incidence of 3.5 in 100.000 live births, MPS belong to the orphan diseases [3]. The most frequent ocular complications are: corneal clouding, glaucoma, ocular hypertension, retinal degeneration and optic nerve swelling or atrophy [4].

The corneal clouding affects most notably MPS of types I, IV and VI. Histopathological examinations of the corneas explanted from MPS patients revealed intracellular and extracellular accumulations of the GAG not only in the corneal stroma, but also in epithelium close to the attenuated Bowman´s layer [5,6]. Using in vivo confocal microscopy, intracellular and extracellular hyperreflective microdeposits are detectable from the level of basal epithelium to the level of Descemet membrane [7,8].

Scheimpflug imaging is a common technique used to evaluate anterior and posterior corneal surface, corneal thickness, corneal density as well as morphology of the anterior chamber and the lens. Aberrometric and topometric indices have been introduced to describe the physiological corneal shape and subclinical ectatic changes [9]. So far, limited information is available concerning the shape of MPS—affected corneas. Although the central corneal thickness (CCT) in MPS varies strongly, its average thickness seems to be comparable to healthy controls [10,11]. Corneal density is increased when compared to healthy controls and strongly correlates with the grade of corneal clouding [11,12]. In a previous study, we analyzed biomechanical indices of corneas affected with lysosomal storage diseases and their influence on intraocular pressure measurements [11]. Corneal hysteresis (CH) and corneal resistance factor (CRF) assessed with Ocular Response Analyzer (ORA, Reichert Inc., Buffalo, NY) strongly correlated with the grade of corneal opacity. Corneal-compensated intraocular pressure calculated on the basis of CH revealed less correlation with corneal opacity than Goldmann applanation tonometry. In the current study, we aimed to evaluate an influence of the MPS-related opacity on the topometric and aberrometric characteristics of the cornea.

Although several studies focused on the microstructural and biomechanical corneal changes in MPS, the impact of these changes on corneal shape and aberrations remains unanswered. We hypothesize that irregularities of the anterior and posterior corneal surfaces as well as HOAs in MPS-affected corneas relevantly differ from healthy controls, similar to the findings in corneal dystrophies. We think that altered tomographic characteristics could be influencing the optical quality of the eye in addition to the decreased corneal transparency.

This first prospective assessment of the tomography of the MPS-affected corneas aims to report to what extent the anterior and posterior corneal shapes as well as HOAs differ between MPS patients and healthy controls.

## Materials and methods

As described in detail previously [11], this observational clinical study was carried out in accordance with the Declaration of Helsinki. Ethics approval was obtained from the Ethics committee of Rhineland-Palatinate, Germany. All MPS patients were referred from the Department of Lysosomal Storage Disorders of the Children´s Hospital, Mainz University Medical Center. The diagnosis of MPS was confirmed by molecular genetic studies. All patients and volunteers were evaluated between September 2012 and November 2013 at the Department of Ophthalmology University Medical Center of the Johannes Gutenberg University Mainz. Written informed consent was obtained from all participants and/or their parents.

**Fig 1:**
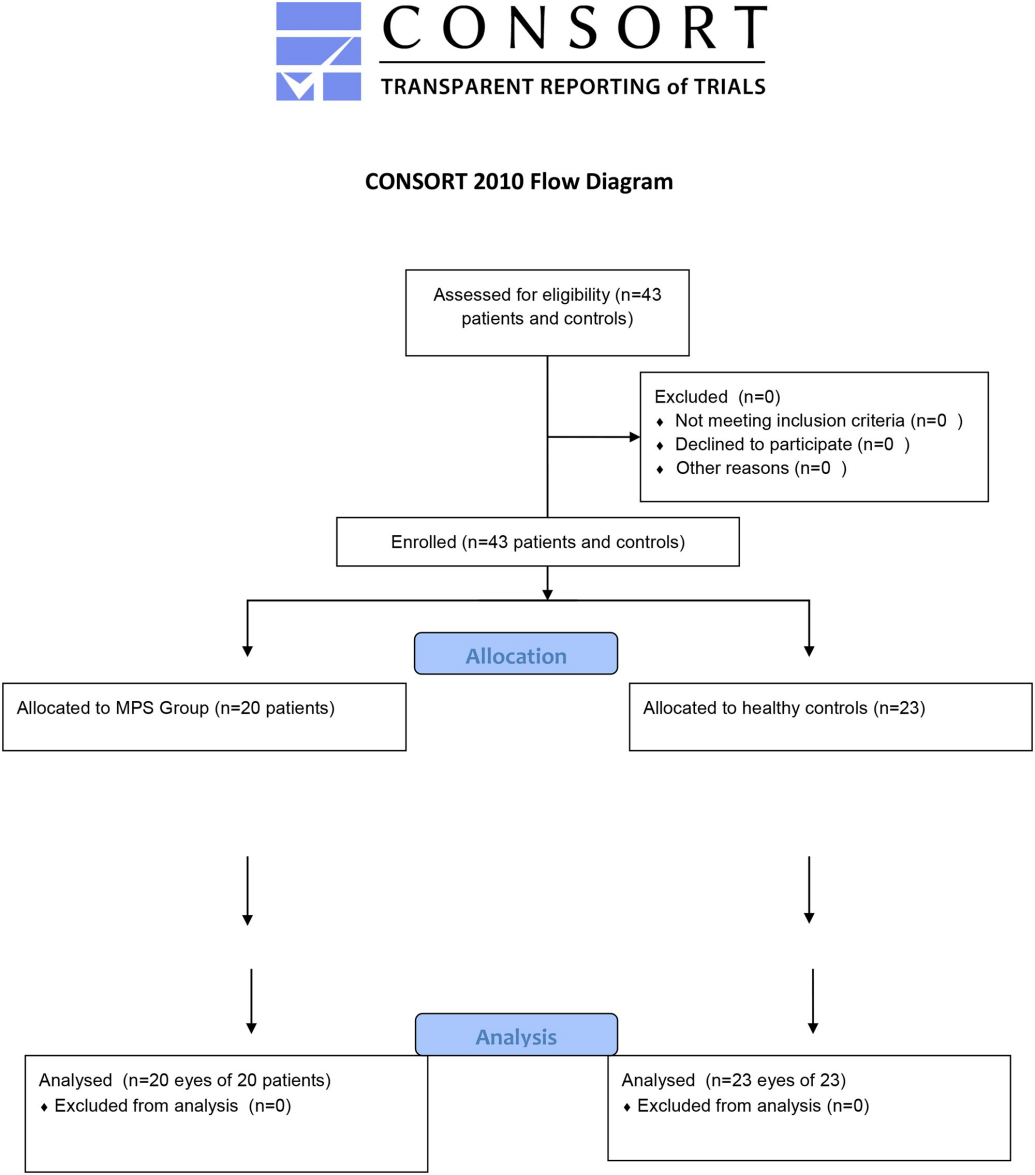
Consort 2010 Flow Diagram.

The Consort 2010 Flow Diagram was shown in Fig 1. The study protocol was attached as S2 Fig.

**ClinicalTrials.gov Identifier:** NCT01695161.

### Patients

The patients evaluated in this study are part of the cohort from our previous trial [11]. As in subjects with the most advanced corneal opacity of grade 4 Scheimpflug imaging of the posterior corneal surface is difficult, we excluded these patients from this substudy. The influence of the biomechanical characteristics of the MPS-affected corneas on the IOP measurements in patients in the current study has been reported previously [11].

The inclusion criteria for all groups were: ≥ 12 years of age and minimal visual acuity of object fixation. Group 1: MPS type I, II, IV or VI with or without corneal clouding (grade 1–3). Group 2: age-matched healthy controls.

The exclusion criteria in both groups were: history of refractive surgery; history of corneal transplantation; history of intraocular surgery with exception of uncomplicated cataract surgery longer than 3 months prior to the study visit; inflammation; corneal pathology other than corneal clouding in MPS patients; corneal opacity grade 4 in the MPS group.

### Examinations

As described in detail previously [11], all patients underwent an assessment of the best-corrected visual acuity (BCVA) with Snellen charts, a slit lamp examination with assessment of the grade of corneal opacity and central fundus. Grading of corneal clouding in MPS patients was done according to Couprie et al. as follows: grade 1 –no corneal clouding visible; grade 2 –mild corneal clouding, still allowing good visibility of details of the anterior chamber, iris and retina; grade 3 –moderate corneal clouding with partial masking of anterior chamber and iris details as well as reduced fundus view; grade 4 –severe corneal clouding without view on anterior chamber and posterior chamber of the eye [13].

Pentacam (Oculus Pentacam HR Typ 70900, Fa. Oculus, Weimar, Germany) is a combination of slit illumination and Scheimpflug camera, which rotate together around the eye. Since cells are not completely transparent, they scatter light and thus produce a sectional image that is recorded by a side-mounted camera. This camera is oriented in Scheimpflug configuration so that a completely focused image from the anterior corneal surface to the back surface of the lens is created. The average of three measurements was calculated [11,14]. Pentacam allows quantification of corneal surface regularity as computed indices, namely: index of surface variance (ISV), index of vertical asymmetry (IVA), index of height asymmetry (IHA), index of height decentration (IHD), keratoconus index (KI) and central keratoconus index (CKI) (Table 1). In addition, mathematical computation leads to quantitative description of corneal surface properties in Zernike indices for both anterior and posterior corneal surface. Vertical trefoil (Z_3;-3_), horizontal trefoil (Z_3;3_), vertical coma (Z_3;-1_), horizontal coma (Z_3;1_) and spherical aberration (Z_4;0_) were included.

**Table 1:** Description of topometric, aberrometric and biomechanical corneal parameters analyzed in our study.

**Topometric parameters (Pentacam)**	
**IVA**	Index of vertical asymmetry
**ISV**	Index of surface variance
**IHD**	Index of height decentration
**IHA**	Index of height asymmetry
**CKI**	central keratoconus index
**KI**	keratoconus index
**ACV**	anterior chamber volume
**ACD**	anterior chamber depth
**CV**	corneal volume
**CCT**	minimum corneal thickness (pachymetrie)
**Density**	corneal density
**Aberrometric parameters (Pentacam)–Higher order aberrations**	
**Z**_**3; 1**_	3rd order horizontal coma aberration at the front
**Z**_**3; -1**_	3rd order vertical coma aberration at the back
**Z**_**3; 3**_	3rd order horizontal trefoil aberration at the front
**Z**_**3; -3**_	3rd order vertical trefoil aberration at the back
**Z**_**4; 0**_	Spherical aberration
**Asph. Q**	Asphericity coefficient
**Biomechanical parameters (ORA)**	
**CRF**	Corneal resistance factor
**CH**	Corneal hysteresis

ORA (Reichert Inc., Buffalo, NY) utilizes a visco-elastic structure of the human corneal tissue in a dynamic bi-directional applanation process. The difference in inward and outward pressure values is called corneal hysteresis (CH). Calculated on the basis of the measured CH, the ORA provides further parameter—the corneal resistance factor (CRF) [11,15]. According to our study protocol, three measurements were taken and the value with the best wave-score (ws) was taken for the statistical analysis.

The description of topometric, aberrometric and biomechanical parameters analyzed in our study is shown in Table 1.

### Statistics

This is an explorative study analysing properties of corneal shape in MPS patients and healthy controls. The included data is based on a previously prospective case control study with the primary outcome as agreement between ccIOP and GAT in MPS, Fabry and healthy controls (ClinicalTrials.gov Identifier: NCT01695161) [11]. Sample size calculation revealed 25 subjects per group. In this secondary data analysis, subjects with sufficient Scheimpflug imaging were included. The eye with better quality Scheimpflug imaging was included; if both eyes showed equal quality index, one eye was determined according to a randomization list.

For descriptive analysis concerning the continuous and non-normally distributed values, the median, 25% and 75% percentiles, minimum and maximum values were calculated. For normally distributed values, the mean values and standard deviations were calculated. Mann-Whitney-U test was used to compare measurements of MPS and healthy controls.

Spearman´s correlation coefficient was performed to assess correlation between corneal shape parameters and biomechanical parameters of the cornea.

Raw data file was attached as S3 Fig.

## Results

### MPS

20 eyes of 20 MPS patients (13 males and 7 females), aged 29.7 ± 11.6 years were enrolled in this study. This group consisted of 3 MPS I, 2 MPS II, 7 MPS IV, 8 MPS VI patients. 13 patients were treated with enzyme replacement therapy (ERT). Thirteen eyes presented mild corneal clouding of grade 2, followed by 5 eyes with moderate corneal clouding of grade 3. Two corneas were clear (grade 1).

### Healthy controls

23 eyes of 23 healthy controls (7 males and 16 females), aged 33.1 ± 9.4 years, range 22.8–58.2 years) were enrolled in this study.

Statistic parameters of anterior chamber, corneal volume, corneal thickness and density in both groups are presented in Table 2. The anterior chamber volume and anterior chamber depth were decreased, whereas the corneal volume and corneal density were increased in MPS compared to healthy controls. The mean minimal corneal thickness was slightly lower in MPS. The biomechanical measurements CH and CRF were significantly higher in the MPS group.

**Table 2:** Topometric and biomechanical parameters of anterior chamber and cornea in healthy controls and mucopolysaccharidosis (MPS). Anterior chamber volume [μL], anterior chamber depth [mm], cornea volume [mm^3^], minimal corneal thickness [μm], corneal density [0 = no clouding, 100 = tissue completely opaque], corneal hysteresis [mmHg] and corneal resistance factor [mmHg].

	Healthy		MPS	
	Median	Interquartile range	Median	Interquartile range
**Anterior chamber volume**	176.5	39.0	111.0	55.0
**Anterior chamber depth**	3.74	0.37	3.04	0.52
**Corneal volume**	60.1	7.13	60.9	10.45
**Minimal corneal thickness**	530.5	39.5	521.0	69.0
**Corneal density**	23.28	7.68	31.02	50.63
**Corneal hysteresis**	10.95	3.1	13.35	5.4
**Corneal resistance factor**	10.85	2.8	12.5	5.6

Mean values, standard deviations and p-values of topometric and aberrometric parameters for both study groups are shown in Table 3. The topometric indices of corneal asymmetry such as IVA, ISV, IHD, IHA were significantly higher in MPS patients compared to healthy controls. The keratoconus indices (KI, CKI) did not differ between study groups.

**Table 3:** Median, interquartile range and p-values of topometric, aberrometric and biomechanical parameters in mucopolysaccharidosis (MPS) and healthy controls. For abbreviations please see Table 1.

	**Healthy**	**MPS**	**p-value**
**IVA**	0.14 (0.09)	0.26 (0.21)	**<0.0001**
**ISV**	15.0 (6.0)	28.5 (21.0)	**<0.0001**
**IHD**	0.01 (0.005)	0.02 (0.01)	**0.01**
**IHA**	3.30 (3.4)	7.35 (5.4)	**0.002**
**CKI**	1.0 (0.01)	1.01 (0.02)	**0.01**
**KI**	1.03 (0.02)	1.04 (0.05)	0.05
**Rmin**	7.49 (0.47)	7.55 (0.66)	0.96
**Corneal front surface**			
**Z**_**3; 1**_	-0.13 (0.25)	-0.08 (0.35)	0.60
**Z**_**3; -1**_	-0.19 (0.18)	-0.10 (0.68)	0.66
**Z**_**3; 3**_	-0.03 (0.12)	0.00 (0.17)	0.45
**Z**_**3; -3**_	0.01 (0.10)	0.05 (0.12)	0.25
**Z**_**4; 0**_	0.53 (0.13)	0.39 (0.24)	**<0.0001**
**Asph. Q**	-0.20 (0.08)	-0.44 (0.21)	**<0.0001**
**Corneal back surface**			
**Z**_**3; 1**_	0.17 (0.41)	0.96 (1.23)	**0.001**
**Z**_**3; -1**_	-0.34 (0.44)	-0.29 (1.00)	0.49
**Z**_**3; 3**_	0.11 (0.43)	0.38 (0.82)	0.16
**Z**_**3; -3**_	0.01 (0.24)	0.04 (0.70)	0.93
**Z**_**4; 0**_	1.25 (0.45)	1.68 (0.53)	**0.009**
**Asph. Q**	-0.15 (0.22)	0.04 (0.44)	**<0.0001**

The higher order aberrations such as: spherical aberration (Z (4; 0)) at the front and at the back surface; asphericity coefficient (Asph. Q) at the front and at the back surface differed significantly between the study groups with lower spherical aberration at the front surface and higher spherical aberration at the back surface.

### Correlations

The Spearman correlation coefficients were evaluated between topometric/aberrometric measurements and corneal biomechanical parameters (CH, CRF, corneal density and the grade of corneal opacity) in both study groups. Those parameters differing between MPS patients and healthy controls are shown in Table 4.

**Table 4:** Spearman correlation coefficients between topometric/aberrometric and biomechanical parameters in mucopolysaccharidosis patients (MPS). Those parameters being different between MPS patients and healthy controls were chosen. Rho—Spearman’s rank correlation coefficient. For abbreviations please see Table 1.

MPS	Corneal density		Corneal opacity		Corneal hysteresis		Corneal resistance factor	
	rho	p-value	rho	p-value	rho	p-value	rho	p-value
**IVA**	**0.72**	0.001	**0.54**	0.02	0.36	0.14	0.46	0.06
**ISV**	**0.52**	0.03	**0.52**	0.03	0.37	0.13	**0.53**	0.03
**IHD**	**0.69**	0.002	**0.48**	0.046	0.35	0.15	0.46	0.05
**IHA**	**0.57**	0.01	**0.57**	0.01	0.34	0.17	0.40	0.10
**Spherical aberration Z**_**4; 0**_ **(front surface)**	-0.02	0.93	-0.13	0.60	0.08	0.76	-0.12	0.65
**Asphericity (Q) (front surface)**	0.06	0.81	-0.06	0.83	0.27	0.28	0.10	0.68
**Spherical aberration Z**_**4; 0**_ **(back surface)**	0.44	0.07	0.33	0.18	**0.54**	0.02	**0.62**	0.006
**Asphericity (Q) (back surface)**	0.39	0.11	0.38	0.12	0.29	0.25	0.32	0.20

Grade of the MPS-associated corneal opacity correlated strongly with topometric parameters [ISV (rho = 0.52), IVA (rho = 0.54), IHA (rho = 0.57), IHD (rho = 0.48)], 3rd order horizontal trefoil aberration at the back [Z (3; 3): rho = 0.62] and at the front surface [Z (3; 3): rho = 0.48]. Correlation between the MPS-associated corneal opacity and biomechanical parameters was positive, but not statistically significant [CH (rho = 0.43, p = .08), CRF (rho = 0.44, p = .07)].

Density of the MPS-affected corneas correlated significantly with topometric parameters [ISV (rho = 0.52), IVA (rho = 0.72), IHA (rho = 0.57), IHD (rho = 0.69)], 3rd order horizontal trefoil aberration at the back [Z (3; 3): rho = 0.62] and at the front surface [Z (3; 3): rho = 0.48] as well as with the biomechanical properties [CH (rho = 0.55), CRF (rho = 0.57)].

Furthermore, in the MPS group CRF correlated significantly with ISV (rho = 0.53). Both, CRF and CH correlated with the spherical aberration at the back surface (rho = 0.62 and rho = 0.54 respectively).

In healthy controls: There were no significant correlations between topometric and biomechanical parameters in the control group. CRF correlated with spherical aberration at the back surface (rho = 0.54), whereas CH correlated with spherical aberration at the front (rho = 0.43) and at the back surface (rho = 0.58).

## Discussion

To the best of our knowledge, this is the first study evaluating corneal shape as well as higher order aberrations in mucopolysaccharidosis patients. The wavefront analysis using Pentacam revealed significant asymmetry of the corneal surface as well as altered higher order aberrations in MPS patients in comparison to healthy controls. The quantification of these somehow expected results gives origin for further studies.

The Pentacam-derived topometric indices such as: index of surface variance, index of vertical asymmetry, index of height asymmetry, index of height decentration, keratoconus index and central keratoconus index, have been developed for the grading and classification of keratoconus as well as post-operative assessment of corneal asymmetry [16]. All these indices apart from keratoconus index were significantly increased in the MPS group. Hashemi *et al*. found index of vertical assymetry to be the best index capable of detecting keratoconus with a cut-off point 0.2 μm [17]. The median index of vertical assymetry value of our MPS patients was 0.26 μm. However, the keratoconus indices and median minimal corneal thickness presented normal values and were comparable between both study groups. None of the MPS patients or healthy probands presented clinical or tomographic signs of keratoconus or ectatic changes. Furthermore, the topometric indices of corneal asymmetry correlated strongly with the grade of corneal opacity and with the corneal density in the MPS group. In the absence of any other clinical or tomographic (normal KI, CKI) signs of ectatic changes we think that the increased values of IVA, ISV, IHA and IHD are the result of microstructural changes in the MPS-affected corneas rather than signs of corneal ectasia. These parameters could be potentially considered as new objective diagnostic and/or follow-up parameters for the evaluation of MPS-related corneal changes.

Not only the topometric indices of corneal asymmetry, but also some higher order aberrations such as spherical aberration and asphericity coefficient at the front and at the back surface differed significantly between MPS and healthy controls. The higher order aberrations are subtle and complex refractive errors, which cannot be corrected with regular eyeglasses or most contact lenses. HOAs comprise about 10% of the eye´s total aberrations. Decrease of contrast sensitivity and different visual symptoms seem to be associated with altered ocular aberrations. Especially spherical aberration was reported to correlate with the low-contrast acuity, monocular diplopia, halos, starburst and glare [18]. Increased HOAs in the lattice and macular corneal dystrophies correlated with poorer visual acuity [1]. Although these results cannot be compared to ours because of different diagnostic methods, we speculate that stromal opacity may not be the only reason for visual impairment associated with MPS. Some visual symptoms reported by MPS-patients may potentially be attributed to altered HOAs. This hypothesis could not be evaluated in the present study and needs to be proven in further studies investigating correlations between the corneal tomography and visual symptoms attributed to altered aberrations such as contrast sensitivity, monocular diplopia, starburst or glare.

The mean minimal corneal thickness and corneal volume did not differ between MPS and healthy controls, whereas anterior chamber depth and volume were decreased in MPS patients. Similar to our previous findings, biomechanical parameters such as corneal density, corneal hysteresis and corneal resistance factor were significantly increased in the MPS group. Interestingly, CH and CRF correlated with the spherical aberration at the back surface in both MPS and healthy subjects. Koc *et al*. also was able to demonstrate a significant correlation between CH and spherical aberration in healthy patients after cataract surgery [19].

In our previous study, we showed to what extent the MPS-related corneal opacity may influence the intraocular pressure measurements performed with different devices. Only two Pentacam parameters such as corneal thickness and density were taken into account in this trial [11]. In the present study, we aimed to evaluate the influence of MPS-related opacity on such sophisticated parameters as the corneal shape and the higher order aberrations. As in subjects with the most advanced corneal opacity of grade 4 Scheimpflug imaging of the posterior corneal surface is difficult, we excluded these patients from this substudy.

One of the limitations by implementing Scheimpflug imaging in MPS patients is the difficulty in adequately positioning the patients in front of the device due to their short stature. The relatively long scanning time needs stable fixation of the fixation mark which otherwise may hamper the quality of image acquisition: poor cooperation due to mental status or severe visual impairment represents a limitation when implementing this method. While Scheimpflug imaging examines the anterior and posterior corneal surface, future studies should incorporate wave front measurements of the eye to evaluate the impact of corneal aberrations on optical quality in MPS patients.

In conclusion, the life span and the quality of life of patients suffering from MPS improved in the last decades. However, the enzyme replacement therapy seems not to improve the ocular outcome [20]. Use of regular spectacles is the most common method for correction of refractive errors. Penetrating keratoplasty is a treatment option reserved for advanced stages of corneal clouding. So far, little was known about more subtle corneal changes such as surface irregularities and ocular aberrations associated with MPS. Better understanding of these phenomena could potentially result in a more sophisticated therapeutical approach (i.e. refractive surgery or usage of special contact lenses instead of spectacles) in this group of patients in the future. The topometric indices of the ocular surface should be considered as potential follow-up parameters in MPS-affected corneas. Influence of the corneal irregularities and aberrations on the visual function should be investigated in more detail in the future studies.

## Supporting information

S1 Fig CONSORT 2010 checklist. (DOCX)

S2 Fig Study protocol. (DOC)

S3 Fig Raw data. (XLSX)
